# Population-based study of recurrent DNA damage response gene variants in breast cancer cases

**DOI:** 10.1007/s10549-025-07634-5

**Published:** 2025-02-26

**Authors:** Anna Tervasmäki, Timo A. Kumpula, Mervi Grip, Susanna Koivuluoma, Meeri Seuranen, Robert Winqvist, Tuomo Mantere, Katri Pylkäs

**Affiliations:** 1 Laboratory of Cancer Genetics and Tumor Biology, Translational Medicine Research Unit, University of Oulu, Aapistie 5A, 90220 Oulu, Finland; 2 Biocenter Oulu and Medical Research Center Oulu, University of Oulu, Oulu, Finland; 3 Department of Surgery, Oulu University Hospital and University of Oulu, Oulu, Finland; 4 Northern Finland Laboratory Centre Oulu, Oulu, Finland

**Keywords:** Breast cancer, Genetics of cancer risk, DNA damage response gene variants, Double heterozygote

## Abstract

**Purpose:**

Several variants in DNA damage response (DDR) genes increase the probability to develop breast cancer and show enrichment in Northern Finland. Here, the population prevalence and risk estimations were refined for sixteen recurrent pathogenic/likely pathogenic DDR gene variants.

**Methods:**

Variant genotyping was performed in 2343 unselected Northern Finnish breast cancer cases and 4607 cancer-free controls, and tumor features and family history of cancer for the carriers were examined.

**Results:**

Based on their prevalence and carrier family history, the studied *BRCA1* and *BRCA2* variants, *PALB2* c.1592delT, and *ATM* c.7570G > C were confirmed as high-risk alleles, whereas *CHEK2* c.1100delC, *MCPH1* c.909_921del, and *RAD50* c.687delT were moderate-risk alleles. *FANCM* c.5101C > T and c.5791C > T did not associate with overall breast cancer risk. Double carriers were significantly more common in cases (0.5%, 11/2343) than controls (0.07%, 3/4601, OR 7.2). The *BRCA1/2* and *PALB2* c.1592delT carrier tumors all had high proliferation rates, *PALB2* c.1592delT associating also with grade 3 tumors (*p* = 0.002). Progesterone receptor (*p* < 0.05) and estrogen receptor positive tumors were enriched in *ATM* c.7570G > C and *CHEK2* c.1100delC carriers, whereas *MCPH1* c.904_916del carriers had a significantly high percentage of multifocal tumors (38%, *p* = 0.001). Moreover, one *FANCM* c.5101C > T homozygote case suffered severe side effects from chemotherapy.

**Conclusion:**

The studied DDR gene variants were present in 9% of the unselected cases. As the presence of germline pathogenic variants can provide additional value for surgical decision-making and affect the choice of oncological treatments, the results promote the benefits of genetic testing as a part of breast cancer diagnostics.

**Supplementary Information:**

The online version contains supplementary material available at 10.1007/s10549-025-07634-5.

## Introduction

Based on familial clustering, it has been estimated that up to 10% of all breast cancers arise from a strong hereditary background. Pathogenic variants in the two major susceptibility genes, *BRCA1* and *BRCA2* explain about 15–20% of the familial cases [1]. Generally, their frequency in unselected breast cancer cases is under 2% [2, 3]. Both genes encode proteins with a crucial role in the DNA damage response (DDR), and cells with clinically relevant *BRCA1*/*2* variants are deficient in homologous recombination repair [4]. Multiple other players from the DDR pathway, including *PALB2*, *ATM*, and *CHEK2*, are also connected to breast cancer predisposition, although with lower risk [5–8]. Besides the globally well-established genes, there are also other founder risk alleles whose prevalence can be limited to certain geographical areas or ethnic populations [9–11].

While the relative risk associated with *BRCA1* and *BRCA2* pathogenic variants can be as high as 20-fold, variants in the moderate-risk genes generally result in two- to fourfold risk [12, 13]. However, specific variants in these genes can also confer high risk; for example *ATM* missense variants, c.7271T > G (p.Val2424Gly) and c.7570G > C (p.Ala2524Pro), have been defined as high-risk alleles with 11-fold enrichment in European and 8.5-fold enrichment in Northern Finnish unselected breast cancer cases, respectively [13, 14]. Also, many other moderate-to-high risk alleles in DDR genes show enrichment in Northern Finland due to ancestral genetic bottlenecks and the population settlement during the history [15]. These include *PALB2* c.1592delT, *MCPH1* c.909_921del and *RAD50* c.687delT founder alleles, the latter two conferring approximately three- to fourfold risk, *PALB2* potentially higher [9, 10, 16, 17]. However, some of these initial findings were based on small cohorts, warranting investigations in larger datasets. Some of the susceptibility alleles have also been associated with specific tumor features. For example, pathogenic variants in *BRCA1* often lead to triple-negative (estrogen [ER], progesterone [PR], and human epidermal growth factor 2 [HER2] receptor negative) breast tumors that commonly overlap with basal-like type [18, 19], whereas *ATM* and *CHEK2* variants associate with ER-positive tumors [20, 21]. Interestingly, *FANCM* is a contradictory DDR gene, since its most common truncating variants, c.5101C > T and c.5791C > T, have only a minor effect on the overall breast cancer risk but associate with triple-negative subtype [21–23].

To obtain population prevalence and more precise risk estimations for the recurrent moderate-to-high risk breast cancer susceptibility alleles, here we genotyped 2343 Northern Finnish unselected breast cancer cases and 4607 cancer-free controls for 16 pathogenic/likely pathogenic variants in *BRCA1*, *BRCA2*, *PALB2, ATM*, *CHEK2*, *FANCM, MCPH1* and *RAD50* DDR genes, which have previously been associated with breast cancer susceptibility in the Finnish population [9, 10, 14, 16, 24–26] or reported as recurrent in breast cancer families in the clinics [27]. Our results provide further evidence for the breast cancer associations, refine the risk for specific subtypes, and show that carriers of two or more susceptibility alleles are enriched in breast cancer cases.

## Materials and methods

### Study cohorts

The breast cancer cohort consisted of 2343 consecutive female cases operated for their breast tumor during the years 2000–2022 at the Oulu University Hospital, Northern Ostrobothnia Hospital District in Finland (age at diagnosis 23–94 years, mean 58 years). The cases were unselected for the family history of cancer and age at disease onset, and all diagnoses were pathologically confirmed. The family history of cancer for the cohort was based on questionnaires and was self-reported by the participants. The clinical features for the tumors were obtained from pathology reports. All cases gave written informed consent to participate in the study. The control group consisted of 4607 geographically matched Northern Finnish cancer-free females: 3849 from hospital biobank of Northern Ostrobothnia Hospital District, Biobank Borealis (age 40–98 years), and 758 from Finnish Red Cross (age 18–66 years).

### Genotyping

The genotyped variants were (1) nine *BRCA1/2* variants present in more than one Northern Finnish breast cancer families [27]: *BRCA1* c.3607C > T, c.3626del, c.4097-2A > G and c.5095C > T, and *BRCA2* c.771_775del, c.3860dupA, c.6275_6276del, c.7480C > T and c.9118-2A > G*,* (2) seven recurrent DDR gene variants with reported enrichment in Finnish breast cancer cases: *PALB2* c.1592delT, *ATM* c.7570G > C, *CHEK2* c.1100delC, *FANCM* c.5101C > T and c.5791C > T, *MCPH1* c.909_921del and *RAD50* c.687delT [9, 10, 14, 16, 24–26]. The DNA for genotyping was derived from peripheral blood. Genotyping in breast cancer and Finnish Red Cross control cohorts was performed with MassARRAY® and iPLEX® Gold (Sequenom, Inc., San Diego, CA) at the Institute for Molecular Medicine Finland (FIMM), high-resolution melt analysis (CFX96 Touch Real-Time PCR Detection System, Bio-Rad, Hercules, CA) using Type-It HRM reagents (Qiagen, Hilden, Germany), or custom TaqMan SNP Genotyping assay (Applied Biosystems, Thermo Fisher Scientific, Waltham, MA) and CFX96 Touch Real-Time PCR Detection System. Genotyping of the Biobank Borealis controls was performed as a part of the FinnGen project using ThermoFisher Axiom custom array v2 (imputed variants not included) [28], and FinnGen reported variant status was confirmed from Biobank retrieved original DNA samples. Verification of the identified variants in all cohorts was done with Sanger sequencing (ABI3500xL, Applied Biosystems, Foster City, CA) in Biocenter Oulu Sequencing Center.

### Statistical analysis

The variant frequencies between cases and controls were compared with *Χ*^2^ test or Fisher’s exact test, and with binary logistic regression for *BRCA1* and *BRCA2* variants. Comparison of the tumor characteristics between carriers and non-carriers was done with *Χ*^2^ test or Fisher’s exact test, and the mean age at diagnosis was compared with Mann–Whitney U test. Benjamini–Hochberg method was applied for multiple comparisons of the tumor characteristics, and after using false discovery rate 0.25, *p* values below 0.05 were considered statistically significant. All reported *p* values were two-sided. Analyses were performed by using IBM SPSS Statistics 29.0 for Windows (IBM Corp. Armonk, NY).

## Results

### Recurrent DDR gene variants associate with breast cancer

Altogether 16 pathogenic/likely pathogenic variants in DDR genes were genotyped (Table 1). Since the selected *BRCA1* and *BRCA2* variants remained rare, they were combined for the analysis (the individual frequencies are presented in Supplementary Table 1). The overall prevalence of the studied *BRCA1/2* variants in the unselected breast cancer cohort was 0.9% (21/2338, of which 7 *BRCA1* and 14 *BRCA2*), which was significantly higher compared to controls (odds ratio [OR] 6.7, 95% confidence interval [CI] 2.24–19.70, *p* < 0.001). For *PALB2* c.1592delT, *ATM* c.7570G > C, *CHEK2* c.1100delC, *MCPH1* c.909_921del and *RAD50* c.687delT the association with breast cancer was also significant, whereas *FANCM* c.5101C > T (OR 0.9, 95% CI 0.57–1.28) and c.5791C > T (OR 0.8, 95% CI 0.43–1.61) remained under statistical significance with slightly lower frequency in cases compared to controls (Table 1).

**Table 1:** Frequencies of the studied variants in Northern Finnish unselected breast cancer cases and controls

Variant	Cohort	Var	%	WT	%	OR	95% CI	*p*
All *BRCA1/2 * variants combined^a^	BC cases	21	0.9%	2317	99.1%	6.65	2.24–19.70	< 0.001^b^
	Controls	4	0.1%	3845^c^	99.9%			
*PALB2* c.1592delT, p.Leu531fs	BC cases	16	0.7%	2326	99.3%	5.27	2.06–13.48	< 0.001
rs180177102, 16-23634953-CA-C	Controls	6	0.1%	4595	99.9%			
*ATM* c.7570G > C, p.Ala2524Pro	BC cases	13	0.6%	2329	99.4%	25.63	3.35–196.05	< 0.001
rs769142993, 11-108331498-G-C	Controls	1	0.02%	4592	99.98%			
*CHEK2 *c.1100delC, p.Thr367fs	BC cases	59^d^	2.5%	2283	97.5%	5.91	3.55–9.84	< 0.001
rs555607708, 22-28695868-AG-A	Controls	20	0.4%	4574	99.6%			
*FANCM* c.5101C > T, p.Gln1701*	BC cases	34^d^	1.5%	2308	98.5%	0.85	0.57–1.28	0.478
rs147021911, 14-45189123-C-T	Controls	74^d^	1.7%	4282	98.3%			
*FANCM *c.5791C > T, p.Arg1931*	BC cases	13	0.6%	2329	99.4%	0.83	0.43–1.61	0.630
rs144567652, 14-45198718-C-T	Controls	29	0.7%	4327	99.3%			
*MCPH1* c.909_921del, p.Arg304fs	BC cases	34	1.5%	2309	98.5%	2.06	1.27–3.34	0.004
rs747489687, 8-6444625-CTTG…-C	Controls	32	0.7%	4470	99.3%			
*RAD50* c.687delT, p.Ser229fs	BC cases	28	1.2%	2314	98.8%	4.61	2.34–9.08	< 0.001
rs760146707, 5-132579996-GT-G	Controls	12	0.2%	4570	99.8%			
Total variant carriers	BC cases	208^e^	8.9%	2126	91.1%			
Total variant carriers (excl*. FANCM*)	BC cases	165^e^	7.1%	2171	92.9%			

Genomic locations are reported in GRCh38

BC: breast cancer, CI: confidence interval, Excl: excluding, Var: variant carrier, OR: odds ratio

^a^*BRCA1* c.3607C > T, c.3626del, c.4097-2A > G and c.5095C > T, and *BRCA2* c.771_775del, c.3860dupA, c.6275_6276del, c.7480C > T and c.9118-2A > G

^b^Logistic regression, otherwise *χ*^2^ or Fisher’s exact test

^c^For *BRCA1* c.4097-2A > G, c.5095C > T and *BRCA2* c.7480C > T variants only Finnish Red Cross controls available

^d^Includes one homozygote

^e^Double carriers are counted only once

Of these genes, pathogenic variants in *BRCA1*, *BRCA2* and *PALB2* have globally been classified as high-risk alleles [1, 29], and high risk has recently been demonstrated for *ATM* c.7570G > C as well [14]. Here, the individual *BRCA1* and *BRCA2* variants remained rare in the unselected cases with the combined OR 6.7. Only the most frequent allele, *BRCA2* c.9118-2A > G (p.Val3040Metfs*20), reached statistical significance individually (6/2343, 0.3%, OR 9.88, 95% CI 1.19–82.11, *p* = 0.014, Supplementary Table 1). Also, *PALB2* c.1592delT (0.7%, OR 5.3, 95% CI 2.06–13.48, *p* < 0.001) and *ATM* c.7570G > C (0.6%, OR 25.6, 95% CI 3.35–196.05, *p* < 0.001) fell in the range of the high-risk alleles, although the confidence intervals especially for *ATM* c.7570G > C were wide (Table 1). The reported family history for the identified *BRCA1*, *BRCA2* and *PALB2* carriers revealed additional breast cancer diagnoses in 1st or 2nd degree relatives for over half of the cases, whereas ovarian cancers were less frequent (Table 2). Several other cancer types were detected in the 1st–3rd degree relatives of almost all *BRCA1*, *BRCA2* and *PALB2* carriers (Supplementary Table 2), the most common ones being stomach (in 10 families), lung (9 families), colorectal (6 families), pancreatic (6 families) and prostate cancer (5 families). Notably, 14% (3/21) of the identified breast cancer affected *BRCA1/2* carriers and 25% (4/16) of the *PALB2* carriers were diagnosed with another cancer by the time of last follow-up, and four of those were in the female reproductive system (uterus, ovaries, and two cervical cancers). For *ATM* c.7570G > C carriers, the family history was less striking, as 5/13 (38%) of the carriers had 1st or 2nd degree relatives with breast cancer, despite the high OR estimate (Table 2, Supplementary Table 2).

**Table 2:** Family history of cancer in *BRCA1/2, PALB2* and *ATM* variant carrier cases

Carriers with	*BRCA1/2* (N = 21)		*PALB2* (N = 16)		*ATM* (N = 13)	
	N	%	N	%	N	%
Other own cancers in addition to breast cancer	3	14%	4	25%	2	15%
≥ 1 Breast cancer cases in 1st–2nd degree relatives	12	57%	11	69%	5	31%
≥ 1 Ovarian cancer cases in 1st–2nd degree relatives	4	19%	1	6%	0	0%
≥ 2 Other cancer cases in 1st–3rd degree relatives	10	48%	9	56%	5	38%
No family history of cancer	1	5%	1	6%	1	8%
No information available	3	14%	0	0%	2	15%

Carriers with the following variants were included: *BRCA1* c.3607C > T, c.3626del, c.4097-2A > G and c.5095C > T; *BRCA2* c.3860dupA, c.6275_6276del, c.7480C > T and c.9118-2A > G; *PALB2* c.1592delT; *ATM* c.7570G > C

*CHEK2*, *MCPH1* and *RAD50* variants all had over 1% prevalence in the case cohort (≥ 0.2% in controls), the most prevalent being *CHEK2* c.1100delC, which is a relatively common moderate-risk allele with an estimated global OR of 2.7 [21]. It showed a significant enrichment in the breast cancer cases (2.5%, OR 5.9, 95% CI 3.55–9.84, *p* < 0.001, Table 1) with a similar prevalence as reported elsewhere in Finland (2.8%) [26]. *MCPH1* c.909_921del and *RAD50* c.687delT alleles have both been reported to be enriched in Northern Finnish breast cancer cases [9, 10, 30], here the former showing a twofold enrichment in cases (1.5%, OR 2.1, 95% CI 1.27–3.34, *p* = 0.004) and the latter over fourfold enrichment (1.2%, OR 4.6, 95% CI 2.34–9.08, *p* < 0.001). Altogether, the combined prevalence of the studied high and moderate-risk variants in the unselected breast cancer cohort was 7.1% (165/2336), and 8.9% (208/2334) when including the *FANCM* variants (c.5101C > T and c.5791C > T with OR < 1) (Table 1).

### DDR gene variants and association with tumor features

Clinical features of the tumors were compared between the DDR variant carriers and non-carriers (Supplementary Table 3). For the *BRCA1/2* variant carriers, the most significant associations were observed for high Ki67 proliferation rate (*p* = 0.0004) and age at diagnosis (*p* = 0.002), the mean age for *BRCA1/2* carriers being 51 years and for non-carriers 58 years. Also, a significant enrichment of triple-negative tumors was seen among *BRCA1* positive cases. Analogous to *BRCA1/2*, high Ki67 expression was observed for *PALB2* c.1592delT carriers (*p* = 0.004) together with a higher amount of grade 3 tumors (*p* = 0.002). For *PALB2*, there was also an association with node positivity (*p* = 0.035). *ATM* c.7570G > C and *CHEK2* c.1100delC carriers had an enrichment of PR- (*p* < 0.05) and ER-positive tumors, whereas *FANCM* c.5101C > T and c.5791C > T carriers had a trend towards triple-negative tumors (non-significant). For *RAD50* c.687delT carriers, there were no associations for any specific tumor features.

In the *MCPH1* c.909_921del carriers, there was a significant enrichment of multifocal tumors (*p* = 0.001). Up to 38% (13/34) of the carriers were diagnosed with more than one invasive tumor focus in one breast (in non-carriers 16%, 316/1718). Furthermore, when studying the *MCPH1* c.909_921del carriers in more detail, a large proportion (13/34) had also *in situ* growths in addition to the invasive tumor, six of these also having the multifocal tumor type.

### Double carriers are more prevalent in breast cancer cases than controls

Genotyping revealed that homozygosity or double (or triple) heterozygosity of the studied variants was significantly enriched in the breast cancer cohort, as eleven (11/2343, 0.5%) affected cases with more than one allele were identified compared to the three (3/4601, 0.07%) in cancer-free female controls (OR 7.2, 95% CI 2.02–25.94, *p* < 0.001). The most common alleles in the double carriers were *CHEK2* c.1100delC (6/11) and *FANCM* c.5101C > T (5/11) (Table 3). The enrichment in cases remained significant (OR 13.8, 95% CI 1.70–112.10, *p* = 0.003), even when excluding *FANCM* variants from the double carrier evaluation. No clinical features were associated with the double carriership status (Supplementary Table 3L).

**Table 3:** Clinical features of the carriers of ≥ 2 DDR gene variants

Case	Variant 1	Variant 2	Age	Morphology	Grade	T	N	M	ER/PR/HER2	Ki67
1	*BRCA1* c.3626del	*MCPH1* c.909_921del	76	Lobular	2	2	0	0	+/−/−	2
2	*BRCA1* c.5095C > T	*CHEK2* c.1100delC	39	Duct	2	1	0	0	+/+/−	2
3	*PALB2* c.1592delT	*RAD50* c.687delT	60	Duct	3	3	1	0	+/+/−	1
4	*MCPH1* c.909_921del	*CHEK2* c.1100delC	48	Duct	2	1	0	0	+/+/−	2
5	*RAD50* c.687delT	*CHEK2* c.1100delC	54	Duct + DCIS	3	4	1	0	−/−/+	3
6	*RAD50* c.687delT	*CHEK2* c.1100delC *FANCM* c.5101C > *T*	87	Duct/inflam	2	4	1	0	+/+/−	1
7	*CHEK2* c.1100delC	*CHEK2* c.1100delC	51	Duct + DCIS	2	1	0	0	+/+/+	3
8	*FANCM* c.5101C > T	*RAD50* c.687delT	45	Duct	3	2	1	0	+/+/−	3
9	*FANCM* c.5101C > T	*CHEK2* c.1100delC	70	Duct	1	1	0	0	+/+/−	1
10	*FANCM* c.5101C > T	*FANCM* c.5791C > T	35	Duct + DCIS	3	1	0	0	−/−/−	3
11	*FANCM* c.5101C > T	*FANCM* c.5101C > T	65	DCIS	3	0	0	0	−/−/−	3
Ctrl 1	*FANCM c*.5101C > T	*FANCM* c.5101C > T	58							
Ctrl 2	*MCPH1* c.909_921del	*FANCM* c.5101C > T	74							
Ctrl 3	*MCPH1* c.909_921del	*PALB2* c.1592delT	27							

Age: age at diagnosis (cases) or at the time of study participation (controls), Ctrl: control, DCIS: ductal carcinoma in situ, Duct: ductal, ER: estrogen receptor, HER2: human epidermal growth factor receptor 2, Inflam: inflammatory, M: metastasis, N: nodes, PR: progesterone receptor, T: tumor

Of the 11 double carrier cases (Table 3), three had a variant in a high-risk gene (*BRCA1* or *PALB2*) combined with a moderate-risk allele (*MCPH1* c.909_921del or *CHEK2* c. 1100delC combined with a *BRCA1* variant, case 1 and 2, respectively, or *PALB2* c.1592delT combined with *RAD50* c.687delT, case 3). There were no prominent features in any of these three double carriers, although case 2 was diagnosed with breast cancer at a relatively young age (39 years).

The other double carriers had 2 (or more) moderate-risk and/or *FANCM* alleles (Table 3). Case 4 with breast cancer at the age of 48 years had *CHEK2* c.1100delC and *MCPH1* c.909_921del alleles, and her father, diagnosed with prostate cancer (age unknown) and lung cancer (84 years), was also identified as a double carrier. Two cases were heterozygous for both *CHEK2* c.1100delC and *RAD50* c.687delT: case 5 was reported to have a profuse amount of *in situ* component in addition to the invasive class T4 tumor, whereas case 6 (carrying also third, *FANCM* c.5101C > T, variant) was diagnosed simultaneously with three ductal/inflammatory tumors in one breast with skin involvement (T4). Case 7 was homozygous for *CHEK2* c.1100delC variants and was diagnosed with invasive ductal tumor and abundant ductal carcinoma *in situ* at the age of 51 years, and had cancer recurrence five years after the initial diagnosis.

There were two cases with biallelic *FANCM* variants. Case 10 with early onset breast cancer (35 years) was identified as a compound heterozygote for *FANCM* c.5101C > T and c.5791C > T, and had triple-negative ductal tumor together with ductal/lobular carcinoma *in situ* and lymphovascular invasion. The other case (case 11) was homozygous for *FANCM* c.5101C > T, had a triple-negative tumor at the age of 65, and suffered from severe fatigue, cytopenia and neutropenic infection during carboplatin/paclitaxel and epirubicin/cyclophosphamide treatments, despite receiving additional leukocyte growth factor. In contrast, case 10 received docetaxel and 5-fluorouracil/epirubicin/cyclophosphamide therapy, developed only a neutropenic tonsillitis but also needed leukocyte growth factor treatment. For the cases 1–9, no unusual treatment-related adverse effects were reported.

## Discussion

We have investigated the prevalence of 16 pathogenic/likely pathogenic recurrent variants in DDR genes, *BRCA1*, *BRCA2*, *PALB2*, *ATM*, *CHEK2*, *FANCM, MCPH1* and *RAD50*, in Northern Finnish unselected breast cancer cases and controls. The results support the reported breast cancer associations for the studied variants, the risk estimations ranging from 5.3-to 25.6-fold for the high-risk alleles (*BRCA1/2* variants, *PALB2* c.1592delT and *ATM* c.7570G > C), and 2.0-to 5.9-fold for the moderate-risk variants (*CHEK2* c.1100delC, *MCPH1* c.909_921del and *RAD50* c.687delT), the indicative division into moderate- and high-risk categories based here on the OR and prevalence in the control cohort (> 0.2% for the moderate-risk variants). Based on the results, *FANCM* c.5101C > T and c.5791C > T variants do not associate with breast cancer at heterozygous state, but the carrier tumors have a trend towards triple-negativity, which is in line with the existing literature [21, 22]. Altogether, the overall prevalence of the moderate-to-high risk recurrent DDR gene variants in the unselected breast cancer cohort was 7.1% (8.9% when including the *FANCM* variants).

The studied recurrent *BRCA1/2* pathogenic variants were enriched in the cases (0.9%) but remained still individually rare and thus explain only a small proportion of breast cancer cases unselected for family history of cancer and age at disease onset. *BRCA2* variants (0.6%) were more prevalent than *BRCA1* variants (0.3%), which is in line with the consortium studies combining cases from several populations [21]. However, in addition to the studied recurrent variants, these two genes harbor also other singleton susceptibility alleles increasing their overall mutation frequency [27]. *PALB2* founder variant c.1592delT was observed in 0.7% of the cases, which is similar to the frequency reported in elsewhere in Finland (0.75%) [26]. Altogether, the recurrent variants in these three main susceptibility genes with cell level mechanistic similarities, and the carriers of which are most likely benefiting from the use of PARP inhibitors [31], were present in total of 1.6% unselected cases. Also, *ATM* c.7570G > C maintained the status of a high-risk allele (0.6% prevalence in breast cancer cases with OR 25.6), although the risk estimation is with wide CIs (3.35–196.05). All tumors were ER- and PR-positive, which is consistent with the previous studies [21]. The *ATM* c.7570G > C allele is enriched in Northern Finland but is rare in other regions of the country [3, 14, 26].

The two moderate-risk alleles, *MCPH1* c.909_921del (1.5% of the cases) and *RAD50* c.687delT (1.2%), are also known to occur specifically in Northern Finland [9, 10, 30], and both showed significant enrichment in cases compared to controls (twofold for *MCPH1* and 4.6-fold for *RAD50,* respectively). Whereas *RAD50* c.687delT variant did not associate with any clinical tumor features, *MCPH1* c.909_921del carriers had a significant enrichment of multifocal tumors (38% vs. 16% in non-carriers). Curiously, multifocal tumors have previously been reported to be more common also in *BRCA2* pathogenic variant carriers (up to 33%, 40/121) [32], although this phenomenon was not as dominant in *BRCA2* carriers in the current, relatively small cohort (3/14, 21%). Other features have also been associated with multifocality, including positive lymph nodes, additional *in situ* cancers and higher proportion of lobular tumors [33], which were also seen among the *MCPH1* c.909_921del carriers with multifocal tumors. A previous functional study using MCF10A breast epithelial cells has shown that this variant at homozygous state results in larger spheroids in 3D cultures, and increased migration and invasion rates [34]. These results support the potential role of truncated MCPH1 in the invasiveness of the disease, plausibly having an effect on the multifocality of the tumors.

When pooling all the currently investigated variants together, the overall frequency of double heterozygotes/homozygotes in the cases was 0.5% (11/2343), whereas only three double carriers were detected in the controls (0.07%, 3/4601). These results support the previously suggested multiplicative risk model, especially for moderate-risk variants, where the second variant confers additional risk [26, 35]. Here, the most common alleles in the double carriers were *CHEK2* c.1100delC and *FANCM* c.5101C > T, both also being the most prevalent variants in the case cohort (2.5% and 1.5%, respectively). Of these *CHEK2* c.1100delC, besides being defined as a moderate-risk allele, has also suggested to be a multiplying risk factor when combined with other susceptibility alleles [21, 36]. Of the identified *CHEK2* c.1100delC carriers, 8.5% (6/59) had more than one of the currently studied DDR gene variants, one of them being homozygous for *CHEK2* c.1100delC. This homozygosity has previously been shown to increase the breast cancer risk to more than fourfold compared to the general population, the risk in homozygous females being more than twice of that in heterozygotes [37].

Although *FANCM* variants did not show an association with the overall breast cancer risk, two biallelic cases were observed. One was identified as compound heterozygote for *FANCM* c.5101C > T and c.5791C > T, and the other was *FANCM* c.5101C > T homozygote. Both had triple-negative tumors with high Ki67 expression. Homozygous or compound heterozygous *FANCM* loss-of-function variants have previously been reported to increase the breast cancer risk, and cause hypersensitivity to chemotherapy and radiation treatments due to acute toxicity [38–40]. This was also reported for the identified *FANCM* c.5101C > T homozygous case, as she suffered from severe side effects during chemotherapy, similar to those recently described for another *FANCM* homozygote [40]. Thus, despite the effect of *FANCM* heterozygosity on breast cancer predisposition remains unproven, it is important to detect breast cancer cases with biallelic *FANCM* variants to avoid treatments with excessive side effects. Since these two stop-gain variants are relatively common in Finland, biallelic cases are expected to occur in the population, in the current study the prevalence being 0.1% in unselected cases.

In conclusion, a relatively high frequency of the recurrent *BRCA1*, *BRCA2*, *PALB2, ATM*, *CHEK2*, *FANCM, MCPH1,* and *RAD50* variants was detected in the unselected breast cancer cohort. This suggests that moderate- and high-risk susceptibility DDR gene variants are prevalent in around 10% of the cases undergoing breast tumor surgery in Northern Finland. Particularly, combinations of these variants were significantly enriched in the case cohort, supporting the multiplicative effect for moderate-risk variants. The presence of germline pathogenic variants can already be taken into account in the choice of oncological treatment; the *BRCA1/2* pathogenic variant carriers benefit from the use of PARP inhibitors, and based on the mechanistic similarities and ongoing trials, *PALB2* carriers could be considered for the same therapeutic regimen [31, 41, 42]. In contrast, treatments for *FANCM* biallelic cases should be tailored carefully to avoid side effects. Germline pathogenic variant status can also provide additional value for surgical decision-making, from breast-conserving surgery to mastectomy, depending on the magnitude of the risk and/or likelihood of cancer recurrence. Pedigrees of *BRCA1/2* and *PALB2* variant carrier cases indicated that breast and other cancer types are often accumulated in these families, providing further evidence for the presence of inherited risk factors. Altogether, current results promote the benefits of genetic testing as a part of breast cancer diagnostics, which can be extended assessing the cancer risk in family members, not only for high-risk pathogenic variants but also for moderate-risk variants and their combinations.

## Supplementary Information

Below is the link to the electronic supplementary material.

Supplementary file1 (PDF 234 KB)

Supplementary file2 (PDF 238 KB)

Supplementary file3 (PDF 800 KB)

## Data Availability

The data that supports the findings of this study are available in the supplementary material of this article. No datasets were generated or analysed during the current study.
