# Supplementary material for: Population-based study of recurrent DNA damage response gene variants in breast cancer cases
Source: Breast Cancer Res Treat. 2025 Feb 26;211(1):195–202. doi: 10.1007/s10549-025-07634-5 (PMC11953123; doi:10.1007/s10549-025-07634-5)
Supplement: Supplementary file 1 — Supplementary file1 (PDF 234 KB) [file 10549_2025_7634_MOESM1_ESM.pdf]

Supplementary Table 1. *BRCA1* and *BRCA2* variant frequencies in breast cancer cases and controls

Variant	Cohort	Var	%	Wild type	%	OR	95% CI	p-value[c]
BRCA1 c.3607C>T (p.Arg1203Ter)	BC cases	1	0.04%	2341	99.96%	NA	NA	0.453
rs62625308	Controls[a]	0	0%	2832	100%			
17-43091924-G-A								
BRCA1 c.3626del (p.Leu1209Ter)	BC cases	3	0.13%	2339	99.87%	4.93	0.51–47.40	0.155
rs80357571	Controls[a]	1	0.03%	3842	99.97%			
17-43091904-TA-T								
BRCA1 c.4097-2A>G	BC cases	2	0.09%	2339	99.91%	NA	NA	1.000
rs80358019	Controls[b]	0	0%	757	100%			
17-43091034-T-C								
BRCA1 c.5095C>T (p.Arg1699Trp)	BC cases	1	0.04%	2342	99.96%	NA	NA	1.000
rs55770810	Controls[b]	0	0%	758	100%			
17-43063931-G-A								
BRCA2 c.771_775del (p.Asn257fs)	BC cases	0	0%	2340	100%	NA	NA	NA
rs80359671	Controls	ND						
13-32331003-ACAAAT-A								
BRCA2 c.3860dupA (p.Asn1287Lysfs)	BC cases	3	0.13%	2340	99.87%	4.92	0.51–47.37	0.156
rs80359406	Controls[a]	1	0.03%	3841	99.97%			
13-32338208-G-GA								
BRCA2 c.6275_6276del (p.Leu2092fs)	BC cases	2	0.09%	2341	99.91%	3.28	0.30–36.16	0.561
rs11571658	Controls[a]	1	0.03%	3836	99.97%			
13-32340629-CTT-C								
BRCA2 c.7480C>T (p.Arg2494Ter)	BC cases	3	0.13%	2338	99.87%	1.70	0.18–16.37	1.000
rs80358972	Controls[b]	0	0%	754	100%			
13-32356472-C-T								
BRCA2 c.9118-2A>G	BC cases	6	0.26%	2337	99.74%	9.88	1.19–82.11	0.014
(p.Val3040Metfs*20), rs81002862	Controls[a]	1	0.03%	3848	99.97%			
13-32380005-A-G								

BC: breast cancer, CI: confidence interval, NA: not available, ND: not analyzed, OR: odds ratio, Var: variant carrier. [a]Biobank Borealis/FinnGen genotyping controls, [b]Finnish Red Cross blood donor controls (variant not available in the FinnGen platform), [c]χ^2 or Fisher's exact test. The genomic locations are reported in GRCh38.

Population-based study of recurrent DNA damage response gene variants in breast cancer cases

Tervasmäki et al.
