# Supplementary material for: Population-based study of recurrent DNA damage response gene variants in breast cancer cases
Source: Breast Cancer Res Treat. 2025 Feb 26;211(1):195–202. doi: 10.1007/s10549-025-07634-5 (PMC11953123; doi:10.1007/s10549-025-07634-5)
Supplement: Supplementary file 2 — Supplementary file2 (PDF 238 KB) [file 10549_2025_7634_MOESM2_ESM.pdf]

Supplementary Table 2. Family history of cancer in *BRCA1, BRCA2, PALB2* and *ATM* variant carrier cases

Case	Variant	Age	Own ca	BC in 1st/2nd	Ovca in 1st/2nd	Other ca in 1-3rd	Other cancer types in the family
**1**	*BRCA1 c.3607C>T*	42	BC	3	0	1	Colorectal
**2**	*BRCA1 c.3626del*	36	BC + ovca	0	0	6	Throat, pancreas, intestine, liver, sarcoma, leukemia
**3**	*BRCA1 c.3626del + MCPH1*	76	BC + melanoma	0	1	2	Liver, unknown
**4**	*BRCA1 c.3626del*	57	BC	NA	NA	NA	NA
**5**	*BRCA1 c.4097-2A>G*	37	BC	2 [+]	0	4	Bile duct, stomach, prostate, leukemia
**6**	*BRCA1 c.4097-2A>G*	47	BC	0	0	0	
**7**	*BRCA1 c.5095C>T + CHEK2*	39	BC	0	0	1	Lung
**8**	*BRCA2 c.3860dupA*	45	BC	4 [+]	1	2	2x brain, prostate
**9**	*BRCA2 c.3860dupA*	53	BC	1	1	0	
**10**	*BRCA2 c.3860dupA*	54	BC	NA	NA	NA	NA
**11**	*BRCA2 c.6275_6276del*	45	BC	5	0	1	Colon
**12**	*BRCA2 c.6275_6276del*	57	BC	1	0	2	2x lung, stomach
**13**	*BRCA2 c.7480C>T*	50	BC	2	0	2	Colorectal, throat
**14**	*BRCA2 c.7480C>T*	58	BC	1	0	4	Colorectal, stomach, ovca, pancreas
**15**	*BRCA2 c.7480C>T*	70	BC + cervical	NA	NA	NA	NA
**16**	*BRCA2 c.9118-2A>G*	40	BC	4	0	2	Prostate, melanoma
**17**	*BRCA2 c.9118-2A>G*	36	BC	3 [+]	0	1	Stomach
**18**	*BRCA2 c.9118-2A>G*	49	BC	3	1	3	Ovca, prostate, lung
**19**	*BRCA2 c.9118-2A>G*	69	BC	1	0	3	Prostate, bile duct, throat
**20**	*BRCA2 c.9118-2A>G*	62	BC	0	0	1	Melanoma
**21**	*BRCA2 c.9118-2A>G*	52	BC	0	0	1	Skin

Case	Variant	Age	Own cancer	BC in $1^{st}/2^{nd}$	Ovca in $1^{st}/2^{nd}$	Other ca in $1\text{-}3^{rd}$	Other cancer types in the family
**1**	*PALB2 c.1592delT*	66	BC	6	0	1	Colorectal
**2**	*PALB2 c.1592delT*	68	BC	4	0	0	
**3**	*PALB2 c.1592delT*	61	BC	3 [+]	0	5	4x stomach, 2x lung, oesophagus
**4**	*PALB2 c.1592delT*	48	BC + cervical	2	0	2	Uterus, lung
**5**	*PALB2 c.1592delT*	52	BC	2	0	1	2x pancreas
**6**	*PALB2 c.1592delT*	68	BC	2	0	1	Stomach
**7**	*PALB2 c.1592delT*	56	BC	1	0	2	Stomach, pancreas, leukemia
**8**	*PALB2 c.1592delT*	49	BC + basalioma	1	0	1	Lung
**9**	*PALB2 c.1592delT*	64	BC	1	0	2	2x lung
**10**	*PALB2 c.1592delT*	60	BC + uterus + ALL	1 [+]	0	0	
**11**	*PALB2 c.1592delT*	55	BC bilat	1	0	2	Stomach
**12**	*PALB2 c.1592delT + RAD50*	60	BC	0	1	10	Pancreas, liver, brain, renal, spine, 2x lung, melanoma, appendix
**13**	*PALB2 c.1592delT*	64	BC	0	0	0	
**14**	*PALB2 c.1592delT*	87	BC + oesophagus	0	0	5	Uterus, stomach, brain
**15**	*PALB2 c.1592delT*	66	BC	0	0	6	Stomach, colorectal, lung, liver, melanoma
**16**	*PALB2 c.1592delT*	49	BC	0	0	3	Pancreas, bone, prostate
**1**	*ATM c.7570G>C*	43+65	Bilat BC	3	0	4	2x lymphoma, lung, leukemia
**2**	*ATM c.7570G>C*	69	BC	2	0	3	Vaginal, lung, renal, breast
**3**	*ATM c.7570G>C*	58	BC	1	0	0	
**4**	*ATM c.7570G>C*	68	BC	1	0	3	3x stomach
**5**	*ATM c.7570G>C*	56	BC	1	0	3	2x lung, colorectal
**6**	*ATM c.7570G>C*	63	BC + papil thyroid	0	0	5	Spine, skin, 2x visceral, bone
**7**	*ATM c.7570G>C*	66	BC	0	0	1	Thyroid
**8**	*ATM c.7570G>C*	78	BC + skin	0	0	1	Unknown
**9**	*ATM c.7570G>C*	46	BC	0	0	1	Stomach
**10**	*ATM c.7570G>C*	57	BC	0	0	1	Lung
**11**	*ATM c.7570G>C*	54	BC	0	0	1	Breast
**12**	*ATM c.7570G>C*	54	BC	NA	NA	NA	
**13**	*ATM c.7570G>C*	55	BC	NA	NA	NA	

[+] at least one case identified as a carrier, age: age at diagnosis, ALL: acute lymphoblastic leukemia, BC: breast cancer, bilat: bilateral, ca: cancer, NA: not available, ovca: ovarian cancer, papil: papillary
