# Supplementary material for: Population-based study of recurrent DNA damage response gene variants in breast cancer cases
Source: Breast Cancer Res Treat. 2025 Feb 26;211(1):195–202. doi: 10.1007/s10549-025-07634-5 (PMC11953123; doi:10.1007/s10549-025-07634-5)
Supplement: Supplementary file 3 — Supplementary file3 (PDF 800 KB) [file 10549_2025_7634_MOESM3_ESM.pdf]

Supplementary Table 3. Clinical features of the tumors associated with DNA damage response gene variants

A. All *BRCA1* and *BRCA2* [a]

Category	Comparisons	N=21 *BRCA1/2* var	% of valid	N=2046 Wild type	% of valid	OR	95% CI	p
**Tumor**								
**1**		11	55%	1200	64%			
**2**		8	40%	584	31%			
**3**		1	5%	68	4%			
**4**	**2, 3 and 4 vs. 1**	0	0%	23	1%	1.46	0.60–3.53	0.484
**Nodus**								
**Neg**		13	62%	1302	64%			
**Pos**	**posit vs. neg**	8	38%	725	36%	1.11	0.46–2.68	1.000
**Metastasis**								
**Neg**		20	95%	1993	98%			
**Pos**	**posit vs. neg**	1	5%	45	2%	2.21	0.29–16.86	0.379
**ER**								
**Neg**		6	30%	269	14%			
**Pos**	**neg vs. posit**	14	70%	1666	86%	2.65	1.01–6.97	0.051
**PR**								
**Neg**		9	45%	431	23%			
**Pos**	**neg vs. posit**	11	55%	1486	78%	2.82	1.16–6.85	0.028
**HER2**								
**Neg**		18	95%	1583	85%			
**Pos**	**posit vs. neg**	1	5%	281	15%	0.31	0.04–2.35	0.340
**Grade**								
**1**		1	5%	373	19%			
**2**		7	37%	914	47%			
**3**	**3 vs. 1 and 2**	11	58%	672	34%	2.63	1.05–6.58	0.049
**Tumor histology**								
**Ductal**		16	76%	1587	78%			
**Lobular**		4	19%	309	15%			
**Other**	**Duct vs. others**	1	5%	139	7%	0.90	0.33–2.48	0.794
**Type**								
**LumA**		13	68%	1414	76%			
**LumB**		0	0%	199	11%			
**HER2 pos**		1	5%	77	4%			
**Triple-neg**	**TN vs. others**	5	26%	160	9%	3.76	1.34–10.58	0.021
**Ki67**								
**Neg**		0	0%	145	8%			
**1+**		4	21%	763	41%			
**2+**	**high vs. low**	3	16%	501	27%			
**3+**	**3 vs. others**	12	63%	455	24%	5.31	2.08–13.56	0.0004
**Multifocal**								
**Yes**		3	14%	325	16%			
**No**	**yes vs. no**	18	86%	1718	84%	0.88	0.26–3.01	1.000
**Bilateral**								
**Yes**		0	0%	50	2%			
**No**	yes vs. no	21	100%	1996	98%	NA	NA	1.000
**Mean age at diag.**		51 y		59 y				0.002

B. All *BRCA1* [a]

Category	Comparisons	N=7 *BRCA1* var	% of valid	N=2062 Wild type	% of valid	OR	95% CI	p
**Tumor**								
1		2	29%	1211	64%			
2		4	57%	588	31%			
3		1	14%	68	4%			
4	2, 3 and 4 vs. 1	0	0%	23	1%	4.46	0.86–23.04	0.106
**Nodus**								
Neg		7	100%	1310	64%			
Pos	posit vs. neg	0	0%	733	36%	NA	NA	0.055
**Metastasis**								
Neg		7	100%	2008	98%			
Pos	posit vs. neg	0	0%	46	2%	NA	NA	1.000
**ER**								
Neg		4	57%	271	14%			
Pos	neg vs. posit	3	43%	1679	86%	8.26	1.84–37.11	0.009
**PR**								
Neg		5	71%	435	23%			
Pos	neg vs. posit	2	29%	1497	77%	8.60	1.66–44.50	0.008
**HER2**								
Neg		7	100%	1596	85%			
Pos	posit vs. neg	0	0%	282	15%	NA	NA	0.603
**Grade**								
1		0	0%	374	19%			
2		2	33%	920	47%			
3	3 vs. 1 and 2	4	67%	680	34%	3.81	0.70–20.83	0.191
**Tumor histology**								
Ductal		5	71%	1598	78%			
Lobular		1	14%	313	15%			
Other	Duct vs. others	1	14%	140	7%	0.71	0.14–3.67	0.654
**Type**								
LumA		3	43%	1426	77%			
LumB		0	0%	199	11%			
HER2 pos		0	0%	78	4%			
Triple-neg	TN vs. others	4	57%	161	9%	14.10	3.13–63.57	0.002
**Ki67**								
Neg		0	0%	145	8%			
1+		0	0%	769	41%			
2+	high vs. low	2	29%	502	27%			
3+	3 vs. others	5	71%	462	25%	7.66	1.48–39.63	0.012
**Multifocal**								
Yes		0	0%	328	16%			
No	yes vs. no	7	100%	1730	84%	NA	NA	0.606
**Bilateral**								
Yes		0	0%	50	2%			
No	yes vs. no	7	100%	2012	98%	NA	NA	1.000
**Mean age at diag.**		47 y		59 y				0.015

C. All *BRCA2* [a]

Category	Comparisons	N=14 *BRCA2* var	% of valid	N=2056 Wild type	% of valid	OR	95% CI	p
**Tumor**								
1		9	69%	1204	64%			
2		4	31%	589	31%			
3		0	0%	69	4%			
4	2, 3 and 4 vs. 1	0	0%	23	1%	0.79	0.24–2.56	0.78
**Nodus**								
Neg		6	43%	1310	64%			
Pos	posit vs. neg	8	57%	727	36%	2.40	0.83–6.95	0.159
**Metastasis**								
Neg		13	93%	2003	98%			
Pos	posit vs. neg	1	7%	45	2%	3.42	0.44–26.74	0.272
**ER**								
Neg		2	15%	274	14%			
Pos	neg vs. posit	11	85%	1671	86%	1.11	0.24–5.03	0.704
**PR**								
Neg		4	31%	437	23%			
Pos	neg vs. posit	9	69%	1490	77%	1.52	0.46–4.95	0.508
**HER2**								
Neg		11	92%	1593	85%			
Pos	posit vs. neg	1	8%	281	15%	0.52	0.07–4.01	1.000
**Grade**								
1		1	8%	373	19%			
2		5	38%	917	47%			
3	3 vs. 1 and 2	7	54%	678	34%	2.22	0.74–6.63	0.153
**Tumor histology**								
Ductal		11	79%	1595	78%			
Lobular		3	21%	310	15%			
Other	Duct vs. others	0	0%	140	7%	1.03	0.29–3.72	1.000
**Type**								
LumA		10	83%	1419	76%			
LumB		0	0%	199	11%			
HER2 pos		1	8%	77	4%			
Triple-neg	TN vs. others	1	8%	165	9%	0.93	0.12–7.28	1.000
**Ki67**								
Neg		0	0%	145	8%			
1+		4	33%	764	41%			
2+	high vs. low	1	8%	503	27%			
3+	3 vs. others	7	58%	462	25%	4.28	1.35–13.55	0.014
**Multifocal**								
Yes		3	21%	326	16%			
No	yes vs. no	11	79%	1727	84%	1.45	0.40–5.21	0.477
**Bilateral**								
Yes		0	0%	50	2%			
No	yes vs. no	14	100%	2006	98%	NA	NA	1.000
**Mean age at diag.**		52 y		59 y				0.036

D. *PALB2* c.1592delT (p.Leu531fs), rs180177102, 16-23634953-CA-C

Category	Comparisons	N=16 *PALB2* var	% of valid	N=2054 Wild type	% of valid	OR	95% CI	p
**Tumor**								
**1**		7	47%	1208	64%			
**2**		7	47%	585	31%			
**3**		1	7%	68	4%			
**4**	**2, 3 and 4 vs. 1**	0	0%	23	1%	2.04	0.74–5.66	0.182
**Nodus**								
**Neg**		6	34%	1311	64%			
**Pos**	**posit vs. neg**	10	63%	725	36%	3.01	1.09–8.33	0.035
**Metastasis**								
**Neg**		15	94%	2002	98%			
**Pos**	**posit vs. neg**	1	6%	45	2%	2.97	0.38–22.94	0.304
**ER**								
**Neg**		3	20%	273	14%			
**Pos**	**neg vs. posit**	12	80%	1671	86%	1.53	0.43–5.46	0.457
**PR**								
**Neg**		7	47%	433	23%			
**Pos**	**neg vs. posit**	8	53%	1493	78%	3.02	1.09–8.37	0.055
**HER2**								
**Neg**		13	87%	1593	85%			
**Pos**	**posit vs. neg**	2	13%	279	15%	0.88	0.20–3.91	1.000
**Grade**								
**1**		0	0%	374	19%			
**2**		4	27%	919	47%			
**3**	**3 vs. 1 and 2**	11	73%	674	34%	5.28	1.67–16.63	0.002
**Tumor histology**								
**Ductal**		15	94%	1589	78%			
**Lobular**		1	6%	313	15%			
**Other**	**Duct vs. others**	0	0%	141	7%	4.28	0.57–32.53	0.221
**Type**								
**LumA**		10	67%	1421	76%			
**LumB**		2	13%	196	11%			
**HER2 pos**		0	0%	78	4%			
**Triple-neg**	**TN vs. others**	3	20%	163	9%	2.6	0.73–9.31	0.141
**Ki67**								
**Neg**		0	0%	145	8%			
**1+**		3	20%	767	41%			
**2+**	**high vs. low**	3	20%	501	27%			
**3+**	**3 vs. others**	9	60%	459	25%	4.62	1.64–13.04	0.004
**Multifocal**								
**Yes**		2	13%	327	16%			
**No**	**yes vs. no**	14	88%	1724	84%	0.75	0.17–3.33	1.000
**Bilateral**								
**Yes**		1	6%	49	2%			
**No**	yes vs. no	15	94%	2005	98%	2.73	0.35–21.06	0.325
**Mean age at diag.**		60 y		58 y				0.587

E. *ATM* c.7570G>C (p.Ala2524Pro), rs769142993, 11-108331498-G-C

Category	Comparisons	N=13 *ATM* var	% of valid	N=2057 Wild type	% of valid	OR	95% CI	p
**Tumor**								
**1**		10	77%	1204	64%			
**2**		3	23%	590	31%			
**3**		0	0%	69	4%			
**4**	**2, 3 and 4 vs. 1**	0	0%	23	1%	0.53	0.15–1.93	0.398
**Nodus**								
**Neg**		9	69%	1309	64%			
**Pos**	**posit vs. neg**	4	31%	730	36%	0.80	0.25–2.60	0.781
**Metastasis**								
**Neg**		13	100%	2004	98%			
**Pos**	**posit vs. neg**	0	0%	46	2%	NA	NA	1.000
**ER**								
**Neg**		0	0%	276	14%			
**Pos**	**neg vs. posit**	13	100%	1669	86%	NA	NA	0.236
**PR**								
**Neg**		0	0%	440	23%			
**Pos**	**neg vs. posit**	13	100%	1487	77%	NA	NA	0.049
**HER2**								
**Neg**		11	85%	1594	85%			
**Pos**	**posit vs. neg**	2	15%	279	15%	1.04	0.23–4.71	1.000
**Grade**								
**1**		1	8%	373	19%			
**2**		8	62%	915	47%			
**3**	**3 vs. 1 and 2**	4	31%	681	35%	0.84	0.26–2.74	1.000
**Tumor histology**								
**Ductal**		10	77%	1594	78%			
**Lobular**		3	23%	311	15%			
**Other**	**Duct vs. others**	0	0%	141	7%	0.95	0.26–3.45	1.000
**Type**								
**LumA**		11	85%	1419	76%			
**LumB**		2	15%	196	11%			
**HER2 pos**		0	0%	78	4%			
**Triple-neg**	**LumA vs. others**	0	0%	166	9%	1.71	0.38–7.72	0.744
**Ki67**								
**Neg**		1	8%	144	8%			
**1+**		3	23%	767	41%			
**2+**	**high vs. low**	7	54%	497	27%			
**3+**	**3 vs. others**	2	15%	465	25%	0.55	0.12–2.49	0.747
**Multifocal**								
**Yes**		2	15%	327	16%			
**No**	**yes vs. no**	11	85%	1727	84%	0.96	0.21–4.35	1.000
**Bilateral**								
**Yes**		0	0%	50	2%			
**No**	**yes vs. no**	13	100%	2007	98%	NA	NA	1.000
**Mean age at diag.**		60 y		58 y				0.711

F. *CHEK2* c.1100delC (p.Thr367fs), rs555607708, 22-28695868-AG-A

Category	Comparisons	N=59 *CHEK2* var	% of valid	N=2012 Wild type	% of valid	OR	95% CI	p
**Tumor**								
1		37	65%	1177	64%			
2		16	28%	577	31%			
3		1	2%	68	4%			
4	2, 3 and 4 vs. 1	3	5%	20	1%	0.96	0.55–1.66	0.890
**Nodus**								
Neg		35	60%	1283	64%			
Pos	posit vs. neg	23	40%	711	36%	1.19	0.70–2.02	0.579
**Metastasis**								
Neg		56	97%	1961	98%			
Pos	posit vs. neg	2	3%	44	2%	1.59	0.38–6.73	0.373
**ER**								
Neg		3	5%	273	14%			
Pos	neg vs. posit	53	95%	1630	86%	0.34	0.11–1.09	0.076
**PR**								
Neg		5	9%	436	23%			
Pos	neg vs. posit	51	91%	1449	77%	0.33	0.13–0.82	0.014
**HER2**								
Neg		44	82%	1561	85%			
Pos	posit vs. neg	10	19%	272	15%	1.30	0.65–2.62	0.561
**Grade**								
1		7	13%	367	19%			
2		29	53%	894	46%			
3	3 vs. 1 and 2	19	35%	666	35%	1.00	0.57–1.76	1.000
**Tumor histology**								
Ductal		47	80%	1558	78%			
Lobular		11	19%	303	15%			
Other	Duct vs. others	1	2%	140	7%	1.11	0.59–2.12	0.756
**Type**								
LumA		44	81%	1386	76%			
LumB		8	15%	191	11%			
HER2 pos		2	4%	76	4%			
Triple-neg	LumA vs. others	0	0%	166	9%	1.38	0.69–2.75	0.420
**Ki67**								
Neg		1	2%	144	8%			
1+		20	36%	750	41%			
2+	high vs. low	19	35%	485	26%			
3+	3 vs. others	15	27%	453	25%	1.14	0.63–2.09	0.751
**Multifocal**								
Yes		11	19%	318	16%			
No	yes vs. no	48	81%	1690	84%	1.22	0.63–2.37	0.587
**Bilateral**								
Yes		2	3%	48	2%			
No	yes vs. no	57	97%	1964	98%	1.44	0.34–6.05	0.652
**Mean age at diag.**		56 y		58 y				0.160

G. *FANCM* c.5101C>T and c.5791C>T combined

Category	Comparisons	N=46* *FANCM* var	% of valid	N=2024 Wild type	% of valid	OR	95% CI	p
**Tumor**								
**1**		22	56%	1193	64%			
**2**		15	38%	576	31%			
**3**		1	3%	68	4%			
**4**	**2, 3 and 4 vs. 1**	1	3%	22	1%	1.38	0.73–2.63	0.399
**Nodus**								
**Neg**		32	73%	1286	64%			
**Pos**	**posit vs. neg**	12	27%	721	36%	0.67	0.34–1.31	0.268
**Metastasis**								
**Neg**		43	98%	1973	98%			
**Pos**	**posit vs. neg**	1	2%	45	2%	1.02	0.14–7.57	1.000
**ER**								
**Neg**		8	19%	267	14%			
**Pos**	**neg vs. posit**	34	81%	1649	86%	1.45	0.67–3.17	0.366
**PR**								
**Neg**		12	29%	428	23%			
**Pos**	**neg vs. posit**	29	71%	1471	77%	1.42	0.72–2.81	0.345
**HER2**								
**Neg**		36	88%	1568	85%			
**Pos**	**posit vs. neg**	5	12%	277	15%	0.79	0.31–2.02	0.670
**Grade**								
**1**		12	29%	362	19%			
**2**		14	33%	908	47%			
**3**	**3 vs. 1 and 2**	16	38%	669	35%	1.17	0.62–2.19	0.743
**Tumor histology**								
**Ductal**		39	85%	1565	78%			
**Lobular**		4	9%	310	15%			
**Other**	**Duct vs. others**	3	7%	138	7%	1.60	0.71–3.59	0.287
**Type**								
**LumA**		30	73%	1400	76%			
**LumB**		3	7%	196	11%			
**HER2 pos**		2	5%	76	4%			
**Triple-neg**	**TN vs. others**	6	15%	159	9%	1.80	0.75–4.35	0.168
**Ki67**								
**Neg**		1	2%	144	8%			
**1+**		18	43%	752	41%			
**2+**	**high vs. low**	7	17%	496	27%			
**3+**	**3 vs. others**	16	38%	452	25%	1.90	1.01–3.57	0.048
**Multifocal**								
**Yes**		5	11%	323	16%			
**No**	**yes vs. no**	40	89%	1699	84%	0.66	0.26–1.68	0.422
**Bilateral**								
**Yes**		1	2%	49	2%			
**No**	yes vs. no	45	98%	1975	98%	0.90	0.12–6.63	1.000
**Mean age at diag.**		59 y		58 y				0.592

*one compound heterozygote

H. *FANCM* c.5101C>T (p.Gln1701Ter), rs147021911, 14-45189123-C-T

Category	Comparisons	N=34 *FANCM* var	% of valid	N=2036 Wild type	% of valid	OR	95% CI	p
**Tumor**								
**1**		19	63%	1196	64%			
**2**		9	30%	583	31%			
**3**		1	3%	66	4%			
**4**	**2, 3 and 4 vs. 1**	1	3%	22	1%	1.03	0.49–2.18	1.000
**Nodus**								
**Neg**		26	79%	1292	64%			
**Pos**	**posit vs. neg**	7	21%	727	36%	0.48	0.21–1.11	0.099
**Metastasis**								
**Neg**		33	100%	1984	98%			
**Pos**	**posit vs. neg**	0	0%	46	2%	NA	NA	1.000
**ER**								
**Neg**		5	16%	270	14%			
**Pos**	**neg vs. posit**	26	84%	1657	86%	1.18	0.45–3.10	0.793
**PR**								
**Neg**		7	23%	432	23%			
**Pos**	**neg vs. posit**	23	77%	1478	77%	1.04	0.44–2.44	1.000
**HER2**								
**Neg**		29	94%	1576	85%			
**Pos**	**posit vs. neg**	2	7%	279	15%	0.39	0.09–1.64	0.304
**Grade**								
**1**		9	28%	365	19%			
**2**		11	34%	911	47%			
**3**	**3 vs. 1 and 2**	12	38%	674	35%	1.14	0.55–2.34	0.712
**Tumor histology**								
**Ductal**		27	79%	1577	78%			
**Lobular**		4	12%	310	15%			
**Other**	**Duct vs. others**	3	9%	138	7%	1.10	0.47–2.53	0.842
**Type**								
**LumA**		24	77%	1407	76%			
**LumB**		2	7%	196	11%			
**HER2 pos**		0	0%	78	4%			
**Triple-neg**	**TN vs. others**	5	16%	160	9%	2.02	0.77–5.33	0.187
**Ki67**								
**Neg**		1	3%	144	8%			
**1+**		12	39%	758	41%			
**2+**	**high vs. low**	5	16%	498	27%			
**3+**	**3 vs. others**	13	42%	455	25%	2.22	1.08–4.57	0.035
**Multifocal**								
**Yes**		4	12%	324	16%			
**No**	**yes vs. no**	30	88%	1709	84%	0.7	0.25–2.01	0.640
**Bilateral**								
**Yes**		1	3%	49	2%			
**No**	yes vs. no	33	97%	1987	98%	1.23	0.17–9.17	0.567
**Mean age at diag.**		59 y		58 y				0.734

I. *FANCM* c.5791C>T (p.Arg1931Ter), rs144567652, 14-45198718-C-T

Category	Comparisons	N=13 *FANCM* var	% of valid	N=2058 Wild type	% of valid	OR	95% CI	p
**Tumor**								
**1**		4	40%	1211	64%			
**2**		6	60%	586	31%			
**3**		0	0%	69	4%			
**4**	**2, 3 and 4 vs. 1**	0	0%	23	1%	2.68	0.75–9.53	0.183
**Nodus**								
**Neg**		7	58%	1311	64%			
**Pos**	**posit vs. neg**	5	42%	729	36%	1.29	0.41–4.06	0.765
**Metastasis**								
**Neg**		11	92%	2006	98%			
**Pos**	**posit vs. neg**	1	8%	45	2%	4.05	0.51–32.06	0.238
**ER**								
**Neg**		4	33%	272	14%			
**Pos**	**neg vs. posit**	8	67%	1675	86%	3.08	0.92–10.30	0.076
**PR**								
**Neg**		6	50%	435	23%			
**Pos**	**neg vs. posit**	6	50%	1494	77%	3.43	1.10–10.70	0.035
**HER2**								
**Neg**		8	73%	1597	85%			
**Pos**	**posit vs. neg**	3	27%	279	15%	2.15	0.57–8.14	0.219
**Grade**								
**1**		3	27%	371	19%			
**2**		3	27%	920	47%			
**3**	**3 vs. 1 and 2**	5	46%	680	35%	1.58	0.48–5.20	0.528
**Tumor histology**								
**Ductal**		13	100%	1593	78%			
**Lobular**		0	0%	314	15%			
**Other**	**Duct vs. others**	0	0%	141	7%	NA	NA	0.085
**Type**								
**LumA**		6	55%	1424	76%			
**LumB**		1	9%	198	11%			
**HER2 pos**		2	18%	76	4%			
**Triple-neg**	**TN vs. others**	2	18%	164	9%	2.30	0.49–10.74	0.254
**Ki67**								
**Neg**		0	0%	145	8%			
**1+**		6	50%	764	41%			
**2+**	**high vs. low**	2	17%	502	27%			
**3+**	**3 vs. others**	4	33%	464	25%	1.52	0.46–5.07	0.506
**Multifocal**								
**Yes**		1	8%	328	16%			
**No**	**yes vs. no**	11	92%	1727	84%	0.48	0.06–3.72	0.704
**Bilateral**								
**Yes**		0	0%	50	2%			
**No**	yes vs. no	13	100%	2008	98%	NA	NA	1.000
**Mean age at diag.**		58 y		58 y				0.986

J. *MCPH1* c.909_921del (p.Arg304ValfsTer3), rs747489687, 8-6444625-CTTGCAAAGAAATA-C

		N=34		N=2037				
**Category**	**Comparisons**	***MCPH1* var**	**% of valid**	**Wild type**	**% of valid**	**OR**	**95% CI**	**p**
**Tumor**								
**1**		16	57%	1199	64%			
**2**		10	36%	583	31%			
**3**		1	4%	68	4%			
**4**	**2, 3 and 4 vs. 1**	1	4%	22	1%	1.34	0.63–2.84	0.552
**Nodus**								
**Neg**		20	59%	1298	64%			
**Pos**	**posit vs. neg**	14	41%	721	36%	1.26	0.63–2.51	0.589
**Metastasis**								
**Neg**		33	97%	1985	98%			
**Pos**	**posit vs. neg**	1	3%	45	2%	1.34	0.18–9.99	0.538
**ER**								
**Neg**		6	19%	270	14%			
**Pos**	**neg vs. posit**	26	81%	1657	86%	1.42	0.58–3.47	0.440
**PR**								
**Neg**		12	38%	428	22%			
**Pos**	**neg vs. posit**	20	63%	1481	78%	2.08	1.01–4.28	0.054
**HER2**								
**Neg**		26	87%	1580	85%			
**Pos**	**posit vs. neg**	4	13%	277	15%	0.88	0.30–2.53	1.000
**Grade**								
**1**		2	7%	372	19%			
**2**		16	55%	907	46%			
**3**	**3 vs. 1 and 2**	11	38%	675	35%	1.16	0.54–2.47	0.845
**Tumor histology**								
**Ductal**		23	68%	1582	78%			
**Lobular**		9	27%	305	15%			
**Other**	**Lob vs. others**	2	6%	139	7%	2.03	0.94–4.94	0.087
**Type**								
**LumA**		23	77%	1408	76%			
**LumB**		2	7%	196	11%			
**HER2 pos**		2	7%	76	4%			
**Triple-neg**	**LumA vs. others**	3	10%	163	9%	1.02	0.43–2.38	1.000
**Ki67**								
**Neg**		0	0%	145	8%			
**1+**		9	30%	761	41%			
**2+**	**high vs. low**	14	47%	490	26%			
**3+**	**3 vs. others**	7	23%	461	25%	0.92	0.39–2.16	1.000
**Multifocal**								
**Yes**		13	38%	316	16%			
**No**	**yes vs. no**	21	62%	1718	85%	3.37	1.67–6.79	0.001
**Bilateral**								
**Yes**		1	3%	49	2%			
**No**	yes vs. no	33	97%	1988	98%	1.23	0.17–9.17	0.567
**Mean age at diag.**		60 y		58 y				0.405

K. *RAD50* c.687delT (p.Ser229fs), rs760146707, 5-132579996-GT-G

		N=28		N=2042				
**Category**	**Comparisons**	***RAD50* var**	**% of valid**	**Wild type**	**% of valid**	**OR**	**95% CI**	**p**
**Tumor**								
**1**		15	60%	1199	64%			
**2**		5	20%	588	31%			
**3**		3	12%	66	4%			
**4**	**2, 3 and 4 vs. 1**	2	8%	21	1%	1.18	0.53–2.65	0.835
**Nodus**								
**Neg**		15	60%	1302	64%			
**Pos**	**posit vs. neg**	10	40%	725	36%	1.2	0.54–2.68	0.678
**Metastasis**								
**Neg**		25	96%	1992	98%			
**Pos**	**posit vs. neg**	1	4%	45	2%	1.77	0.24–13.36	0.446
**ER**								
**Neg**		3	12%	273	14%			
**Pos**	**neg vs. posit**	23	88%	1660	86%	0.79	0.24–2.66	1.000
**PR**								
**Neg**		5	19%	435	23%			
**Pos**	**neg vs. posit**	21	81%	1480	77%	0.81	0.30–2.16	0.816
**HER2**								
**Neg**		22	85%	1584	85%			
**Pos**	**posit vs. neg**	4	15%	277	15%	1.04	0.36–3.04	1.000
**Grade**								
**1**		6	23%	368	19%			
**2**		13	50%	909	46%			
**3**	**3 vs. 1 and 2**	7	27%	679	35%	0.69	0.29–1.66	0.535
**Tumor histology**								
**Ductal**		21	78%	1583	78%			
**Lobular**		5	19%	309	15%			
**Other**	**Duct vs. others**	1	4%	140	7%	1.00	0.40–2.47	1.000
**Type**								
**LumA**		21	81%	1410	76%			
**LumB**		2	8%	196	11%			
**HER2 pos**		2	8%	76	4%			
**Triple-neg**	**LumA vs. others**	1	4%	165	9%	1.30	0.49–3.47	0.654
**Ki67**								
**Neg**		3	12%	142	8%			
**1+**		14	54%	756	41%			
**2+**	**high vs. low**	6	23%	498	27%			
**3+**	**3 vs. others**	3	12%	465	25%	0.39	0.12–1.31	0.167
**Multifocal**								
**Yes**		3	11%	326	16%			
**No**	**yes vs. no**	24	89%	1714	84%	0.66	0.20–2.20	0.790
**Bilateral**								
**Yes**		0	0%	50	2%			
**No**	yes vs. no	28	100%	1992	98%	NA	NA	1.000
**Mean age at diag.**		63 y		58 y				0.090

L. Double carriers

Category	Comparisons	N=11 Double carriers	% of valid	N=2061 Wild type	% of valid	OR	95% CI	p
**Tumor**								
**1**		5	50%	1210	64%			
**2**		2	20%	591	31%			
**3**		1	20%	68	4%			
**4**	**2, 3 and 4 vs. 1**	2	10%	21	1%	1.78	0.51–6.17	0.510
**Nodus**								
**Neg**		7	64%	1311	64%			
**Pos**	**posit vs. neg**	4	36%	731	36%	1.03	0.30–3.51	1.000
**Metastasis**								
**Neg**		11	100%	2007	98%			
**Pos**	**posit vs. neg**	0	0%	46	2%	NA	NA	1.000
**ER**								
**Neg**		3	27%	273	14%			
**Pos**	**neg vs. posit**	8	73%	1676	86%	2.30	0.61–8.73	0.193
**PR**								
**Neg**		4	36%	437	23%			
**Pos**	**neg vs. posit**	7	64%	1494	77%	1.95	0.57–6.70	0.284
**HER2**								
**Neg**		9	82%	1597	85%			
**Pos**	**posit vs. neg**	2	18%	280	15%	1.27	0.27–5.90	0.674
**Grade**								
**1**		1	10%	373	19%			
**2**		5	45%	918	47%			
**3**	**3 vs. 1 and 2**	5	45%	681	35%	1.58	0.48–5.20	0.528
**Tumor histology**								
**Ductal**		10	91%	1596	78%			
**Lobular**		1	9%	313	15%			
**Other**	**Duct vs. others**	0	0%	141	7%	2.85	0.36–22.28	0.473
**Type**								
**LumA**		7	64%	1424	76%			
**LumB**		1	9%	198	11%			
**HER2 pos**		1	9%	77	4%			
**Triple-neg**	**LumA vs. others**	2	18%	164	9%	0.54	0.16–1.85	0.301
**Ki67**								
**Neg**		0	0%	145	8%			
**1+**		3	27%	767	41%			
**2+**	**high vs. low**	3	27%	501	27%			
**3+**	**3 vs. others**	5	45%	464	25%	2.54	0.77–8.35	0.154
**Multifocal**								
**Yes**		2	18%	327	16%			
**No**	**yes vs. no**	9	82%	1730	84%	1.18	0.25–5.47	
**Bilateral**								
**Yes**		0	0%	50	2%			
**No**	yes vs. no	11	100%	2011	98%	NA	NA	1.000
**Mean age at diag.**		57 y		58 y				0.536

[a]*BRCA1* c.3607C>T (p.Arg1203Ter) rs62625308 17-43091924-G-A, *BRCA1* c.3626del (p.Leu1209Ter) rs80357571 17-43091904-TA-T, *BRCA1* c.4097-2A>G rs80358019 17-43091034-T-C, *BRCA1* c.5095C>T (p.Arg1699Trp) rs55770810 17-43063931-G-A, *BRCA2* c.771_775del (p.Asn257fs) rs80359671 13-32331003-ACAAAT-A, *BRCA2* c.3860dupA (p.Asn1287Lysfs) rs80359406 13-32338208-G-GA, *BRCA2* c.6275_6276del (p.Leu2092fs) rs11571658 13-32340629-CTT-C, *BRCA2* c.7480C>T (p.Arg2494Ter) rs80358972 13-32356472-C-T, *BRCA2* c.9118-2A>G (p.Val3040Metfs*20) rs81002862 13-32380005-A-G. CI: confidence interval, Diag: diagnosis, ER: estrogen receptor, HER2: human epidermal growth factor receptor 2, Lum: luminal, Neg: negative, OR: odds ratio, Pos: positive, PR: progesterone receptor, var: variant carrier, y: years. LuminalA was defined as positive ER expression and no HER2 overexpression, luminalB had positive ER and HER2 overexpression, HER2 type as negative ER and PR but with HER2 overexpression, and triple-negative as negative for all three markers. Genomic locations are reported in GRCh38.
